# Elevated plasma cancer antigen-125 level is associated with increased risk of new-onset atrial fibrillation after acute myocardial infarction

**DOI:** 10.3389/fcvm.2025.1718813

**Published:** 2025-12-08

**Authors:** Yuan Fu, Kun Zuo, Mulei Chen, Li Xu, Lin Zhao

**Affiliations:** Heart Center, Beijing Chaoyang Hospital, Capital Medical University, Beijing, China

**Keywords:** acute myocardial infarction, cancer antigen-125, CHA2DS2-VASc score, new-onset atrial fibrillation, risk score model

## Abstract

**Background:**

New-onset atrial fibrillation (NOAF) is the most commonly encountered arrhythmia during the course of acute myocardial infarction (AMI) and is independently associated with a worse prognosis.

**Aims:**

We aimed to validate the discriminatory performance of cancer antigen-125 (CA-125) in predicting post-AMI NOAF in the Chinese population.

**Methods:**

A total of 488 consecutive patients with AMI from 1 January 2020 to 1 January 2022 without a previous history of AF were enrolled in this study. Risk factors for post-AMI NOAF were determined using univariable and multivariable logistic regression analyses. Receiver operating characteristic (ROC) curve analyses were used to evaluate the discrimination performance of different parameters and score models. Area under the curve (AUCs) were compared using *Z* tests.

**Results:**

In total, 48 (9.84%) patients developed post-AMI NOAF during hospitalization. The NOAF group was older and had a higher Killip class, B-type natriuretic peptide level, CA-125 level, left atrial diameter, left ventricular end-systolic diameter, CHA_2_DS_2_-VASc (Congestive heart failure, Hypertension, Age ≥75 years [2 points], Diabetes mellitus, Stroke/transient ischemic attack/thromboembolism history [2 points], Vascular disease, Age 65–74 years, Sex category [female]) score, Global Registry of Acute Coronary Events (GRACE) risk score (RS), and in-hospital mortality, and a lower low-density lipoprotein level, left ventricular ejection fraction, and initial *β*-receptor blocker and angiotensin-converting enzyme inhibitor/angiotensin receptor blocker use (*P* < 0.05 vs. the no-NOAF group for all measures). In the multivariate regression analyses, CA-125 remained an independent risk factor for post-AMI NOAF in different models. In the ROC curve analyses, CA-125 (AUC = 0.753, 95% CI: 0.665–0.84, *P* < 0.001), GRACE RS (AUC = 0.701, 95% CI: 0.593–0.809, *P* = 0.001), and CHA2DS2-VASc score (AUC = 0.644, 95% CI: 0.532–0.757, *P* = 0.014) were valid tools to predict post-AMI NOAF. After *Z* tests, the discriminatory performance of CA-125 was significantly higher than that of the CHA2DS2-VASc score (*P* = 0.038), but not statistically significantly higher than that of the GRACE RS (*P* = 0.353).

**Conclusion:**

An elevated plasma CA-125 level was independently associated with NOAF after AMI, with high discriminatory performance.

## Introduction

New-onset atrial fibrillation (NOAF) is the most common arrhythmia, with an incidence between 4% and 21% in patients with acute myocardial infarction (AMI) and no previous atrial fibrillation (AF) history (1). Post-AMI NOAF is independently associated with increased short- and long-term mortality, and other clinical adverse events, such as stroke and heart failure (HF) (2, 3). Therefore, early and accurate detection of NOAF in the setting of AMI is important to prevent complications and improve the prognosis of patients (4). Previous studies have demonstrated that several clinical factors are related to post-AMI NOAF, such as age, heart rate (HR), high-sensitivity C-reactive protein (hs-CRP) level, and NT-pro-brain natriuretic peptide (NT-proBNP) level (5, 6).

Cancer antigen-125 (CA-125) is a known tumor marker of ovarian cancer and is used to monitor the therapeutic efficacy of anti-cancer treatment (7). Recent studies have shown that an elevated plasma CA-125 level is associated with the presence, development, and recurrence of AF. This may be because inflammation and mechanical stress on the atrial and ventricular walls, important in the development of AF, induce CA-125 production in epithelial cells (7–10). However, the relationship between CA-125 and post-AMI NOAF remains unclear. The aim of this study was to identify the association between CA-125 and NOAF after AMI, and evaluate the discriminatory performance of CA-125 to predict post-AMI NOAF during hospitalization.

## Patients and methods

### Study population

This is an observational retrospective study conducted at Beijing Chaoyang Hospital from 1 January 2020 to 1 January 2022. A total of 492 consecutive patients who were admitted with a diagnosis of AMI and no pre-existing AF were included in this study. Four patients were excluded due to incomplete medical records. Patients were divided into two groups according to their clinical outcomes during hospitalization, i.e., with or without post-AMI NOAF. This study was approved by the Ethics Committee of Beijing Chaoyang Hospital (2020-3-18-31). Informed consent was obtained from all the patients.

AMI was classified as non-ST-segment elevation myocardial infarction (NSTEMI) and ST-segment elevation myocardial infarction (STEMI). Patients with unstable angina were excluded. The diagnosis of AMI was based on the patient’s history, symptoms, electrocardiogram (ECG), and cardiac troponin-I (CTnI) changes according to the third universal definition of myocardial infarction (11). Type 2 myocardial infarction (T2MI) results from a mismatch in oxygen supply and demand, occurring in the absence of coronary plaque disruption or atherothrombosis (11). The pathophysiological mechanisms and management strategies of T2MI are distinct from type 1 myocardial infarction (T1MI), and patients with T2MI were excluded from this study (12, 13).

An initial ECG was conducted within 5 min of the patient’s admission and all the patients received continuous electrocardiography monitoring during hospitalization to detect any arrhythmias. AF was defined as the absence of *P* waves, the presence of fibrillatory waves, an irregular R-R interval, and lasting at least 30 s (14). Post-AMI NOAF was defined as presenting with sinus rhythm on admission with no pre-existing AF history and developing AF at the time of AMI or any time after AMI onset, assessed by ECG or continuous telemonitoring during hospital stay (2). The diagnosis of AF was evaluated by two independent blinded cardiologists.

### Data collection

#### General data collection

Data on the patients' demographics, family and medical history, height, weight, smoking status, and medications were collected on their admission. Weight divided by height squared (kg/m^2^) was used to calculate body mass index (BMI). The Modification of Diet in Renal Disease (MDRD) formula (Chinese version) was used to calculate estimated glomerular filtration rate (eGFR) (15).

Transthoracic echocardiography was conducted in the first 12 h after the patients' admission. Left atrial diameter (LAD) was measured in the parasternal view (M-mode ultrasound). Simpson's method was used to evaluate the left ventricular end-diastolic diameter (LVEDd), left ventricular end-systolic diameter (LVESd), and left ventricular ejection fraction (LVEF). The infarct-related artery (IRA) was identified using coronary angiography (CAG) combined with changes in the ECG.

#### Risk score models

The Global Registry of Acute Coronary Events risk score (GRACE RS) is used for mortality risk stratification of patients with acute coronary syndrome (ACS). It is calculated from eight variables: age, on-admission systolic blood pressure (SBP), HR, Killip class, serum creatinine level, elevated myocardial necrosis biomarkers, ST-segment deviation, and cardiac arrest (16).

The CHADS₂ (Congestive heart failure, Hypertension, Age ≥75 years, Diabetes mellitus, Stroke/transient ischemic attack/thromboembolism history [2 points])​ score and CHA_2_DS_2_-VASc (Congestive heart failure, Hypertension, Age ≥75 years [2 points], Diabetes mellitus, Stroke/transient ischemic attack/thromboembolism history [2 points], Vascular disease, Age 65–74 years, Sex category [female]) score are used for the risk evaluation of ischemic stroke in patients with AF. The CHADS2 score is calculated by assigning 2 points for transient ischemic attack (TIA) or previous stroke, and 1 point each for congestive heart failure (CHF), hypertension (HT), age ≥75 years old, and diabetes mellitus (DM) (17). The CHA2DS2-VASc score is calculated by assigning 2 points for age ≥75 years old and previous TIA or stroke, and 1 point each for CHF, HT, age 65–74 years old, DM, vascular disease, and female gender (18).

#### Laboratory parameter analyses

Venous blood samples were collected on admission and then analyzed using a Dimension RxLMax™ (Siemens Healthcare Diagnostics lnc., Newark, DE, USA) automated analyzer. CA-125 was measured using the chemiluminescent enzyme immunoassay method with a commercially available kit (Roche, Germany). A normal CA-125 level is <35 U/mL. All other biochemical variables were measured by using a Hitachi 7600 (Japan) automatic analyzer.

#### Statistical analysis

The normal distribution of the continuous variables was tested using the Kolmogorov–Smirnov test. Normally distributed variables are presented as mean ± standard deviation (SD) and were analyzed using the Student's *t*-test. Non-normally distributed variables are presented as median (interquartile range) and were analyzed using the Mann–Whitney U test. Dichotomous variables were tested using the *χ*^2^ test and the data are presented as percentages. The risk factors for post-AMI NOAF were identified by univariable analysis and multivariable logistic regression analyses. The receiver operating characteristic (ROC) curve and the area under the curve (AUC) were analyzed to evaluate the discriminatory performance of the different risk scores. The Youden index was calculated as sensitivity + specificity − 1 (19). An AUC < 0.5 represents the absence of predictive ability, while an AUC = 1.0 indicates perfect discriminatory power (20). *Z* tests were used to compare the AUCs of the different models and parameters. A two-sided *P*-value <0.05 was considered statistically significant. All the statistical analyses were conducted using SPSS 24.0 (IBM Corp, Armonk, NY, USA) and MedCalc software (https://www.medcalc.org).

## Results

### Patient baseline characteristics

A total of 488 consecutive patients presenting with a diagnosis of AMI and no pre-existing AF were finally enrolled in this study. The baseline demographic and clinical characteristics of the enrolled patients are shown in Table 1. The median follow-up time of the present study was 10 (6–17) days. Of the study population, 64.14% were male and the mean age was 58.19 ± 11.1 years old. In total, 469 patients underwent coronary angiography during their hospitalization (319 patients underwent emergency coronary angiography). Furthermore, 48 (9.84%) patients developed post-AMI NOAF: 14 (2.87%) cases occurred in the first 24 h after admission and 34 (6.97%) cases occurred after the first 24 h. The patients who developed NOAF during their hospitalization were older and had a higher prevalence of Killip class IV, and had higher CA-125 levels, B-type natriuretic peptide (BNP) levels, LAD, LVESd, CHA2DS2-VASc score, GRACE RS, and in-hospital mortality compared with those who did not develop NOAF (*P* < 0.05 for all measures). In addition, the patients with NOAF had lower low-density lipoprotein (LDL-C) levels and LVEF, and a lower proportion of patients with NOAF had received initial *β*-receptor blocker and angiotensin-converting enzyme inhibitor (ACEI)/angiotensin receptor blocker (ARB) treatment (*P* < 0.05 vs. patients without NOAF for all measures).

**Table 1:** Baseline characteristics of the study population.

Variable	NOAF (*n* = 48)	Without NOAF (*n* = 440)	*P*-value
Age, years	65.67 ± 10.14	57.27 ± 10.9	<0.001
Male, *n* (%)	29 (60.42)	284 (64.55)	0.632
HT, *n* (%)	26 (54.17)	234 (53.18)	0.815
DM, *n* (%)	16 (33.33)	140 (31.82)	0.584
History of MI, *n* (%)	6 (12.5)	61 (13.68)	0.405
History of CHF, *n* (%)	3 (6.25)	41 (9.32)	0.212
History of stroke, *n* (%)	7 (14.58)	54 (12.27)	0.707
Current smoker, *n* (%)	25 (52.08)	237 (53.86)	0.772
BMI, kg/m^2^	24.57 ± 2.52	25.49 ± 3.02	0.27
STEMI, *n* (%)	30 (62.5)	291 (66.14)	0.798
IRA			
LAD	21 (43.75)	196 (44.55)	0.801
LCX	14 (29.17)	144 (32.73)	0.205
RCA	11 (22.92)	88 (20)	0.289
HR at admission, bpm	75.33 ± 12.37	78.13 ± 12.08	0.275
SBP, mmHg	123.19 ± 23.75	127.58 ± 19.52	0.363
DBP, mmHg	63.67 ± 11.42	64.49 ± 12.3	0.284
Killip class			
Class I, *n* (%)	24 (50)	224 (50.91)	0.926
Class II, *n* (%)	14 (29.17)	192 (43.64)	0.109
Class III, *n* (%)	6 (12.5)	18 (4.09)	0.078
Class IV, *n* (%)	4 (8.33)	6 (1.36)	0.03
Medications			
β-RB within 24 h, *n* (%)	23 (47.92)	302 (68.64)	0.033
ACEI/ARB within 24 h, *n* (%)	8 (16.67)	179 (40.68)	0.021
Statin, *n* (%)	47 (97.92)	437 (99.32)	0.813
ESR, mm/h	11 (4–19)	7.5 (4–14)	0.225
hs-CRP, mg/L	23.7 (1.8–99.3)	5.8 (2.15–16.75)	0.073
HbAlc, %	6.23 (5.77–7.5)	6.31 (5.52–7.4)	0.426
BNP (pg/mL)	362 (188–672)	139 (72.25–237.75)	<0.001
WBC, ×10^9^/L	10.01 ± 2.92	10.63 ± 3.18	0.502
RBC, ×10^12^/L	4.05 ± 1.06	4.11 ± 0.93	0.209
Hb, g/L	133 (124–146)	134 (121.3–144)	0.312
D-dimer, mg/L FEU	0.38 (0.23–1.04)	0.31 (0.2–1.11)	0.407
CK-MB, ng/mL	19.03 (10.1–94.27)	23.31 (7.42–131.7)	0.594
CTnI, ng/mL	17.71 (7.28–103.04)	34.02 (6.78–108.43)	0.552
TC, mmol/L	4.05 ± 0.91	4.03 ± 1.13	0.371
HDL-C, mmol/L	0.97 ± 0.27	0.96 ± 0.22	0.831
LDL-C, mmol/L	2.83 ± 1.04	3.06 ± 0.99	0.01
SCR, μmol/L	70.5 (57.1–98.7)	66.2 (58.93–76.9)	0.367
eGFR, mL/min/1.73 m^2^	103.56 ± 46.9	120.2 ± 33.47	0.084
SUA, μmol/L	404.13 ± 132.96	392.77 ± 127.42	0.174
CA-125, U/mL	10.5 (7.4–16.4)	6 (4.3–8.25)	<0.001
Echocardiography parameters			
LVEF (%)	51 (41–60)	61 (51–66)	0.004
LVEDd, mm	49 (47–54)	48 (45–51)	0.051
LVESd, mm	35 (30–39)	31 (28–36)	0.016
LAD	37 (34–42)	35 (33–37)	0.034
IABP, *n* (%)	6 (12.5)	10 (2.27)	0.004
CHADS2 score	2 (1–3)	1 (0–3)	0.142
CHA2S2-VASc score	4 (3–5)	3 (2–4)	0.013
GRACE RS	168 (150–190)	150 (130–166)	0.001
In-hospital mortality, *n* (%)	5 (10.42)	4 (0.91)	<0.001

NOAF, new-onset atrial fibrillation; HT, hypertension; DM, diabetes mellitus; MI, myocardial infarction; CHF, chronic heart failure; BMI, body mass Index; STEMI, ST-segment elevation myocardial infarction; IRA, infarct-related artery; LAD, left anterior descending artery; LCX, left circumflex coronary artery; RCA, right coronary artery; HR, heart rate; SBP, systolic blood pressure; DBP, diastolic blood pressure; ACEI, angiotensin-converting enzyme inhibitor; ARB, angiotensin receptor blocker; ESR, erythrocyte sedimentation rate; hs-CRP, high-sensitivity C-reactive protein; HbA1c, glycosylated hemoglobin; BNP, B-type natriuretic peptide; WBC, white blood cell; Hb, hemoglobin; CK-MB, creatine kinase MB; CTnI, cardiac troponin-I; TC, total cholesterol; HDL-C, high-density lipoprotein cholesterol; LDL-C, low-density lipoprotein cholesterol; SCR, serum creatinine; eGFR, estimated glomerular filtration rate; SUA, serum uric acid; CA-125, cancer antigen-125; LVEF, left ventricular ejection fraction; LVESd, left ventricular end-systolic diameter; LVEDd, left ventricular end-diastolic diameter; LAD, left atrium diameter; IABP, intra-aortic ballon pump; GRACE RS, Global Registry of Acute Coronary Events risk score; FEU, fibrinogen equivalent units.

Data are presented as numbers (%), means (SD), or medians (IQR).

### The discriminatory power of different scores

**Figure 1:**
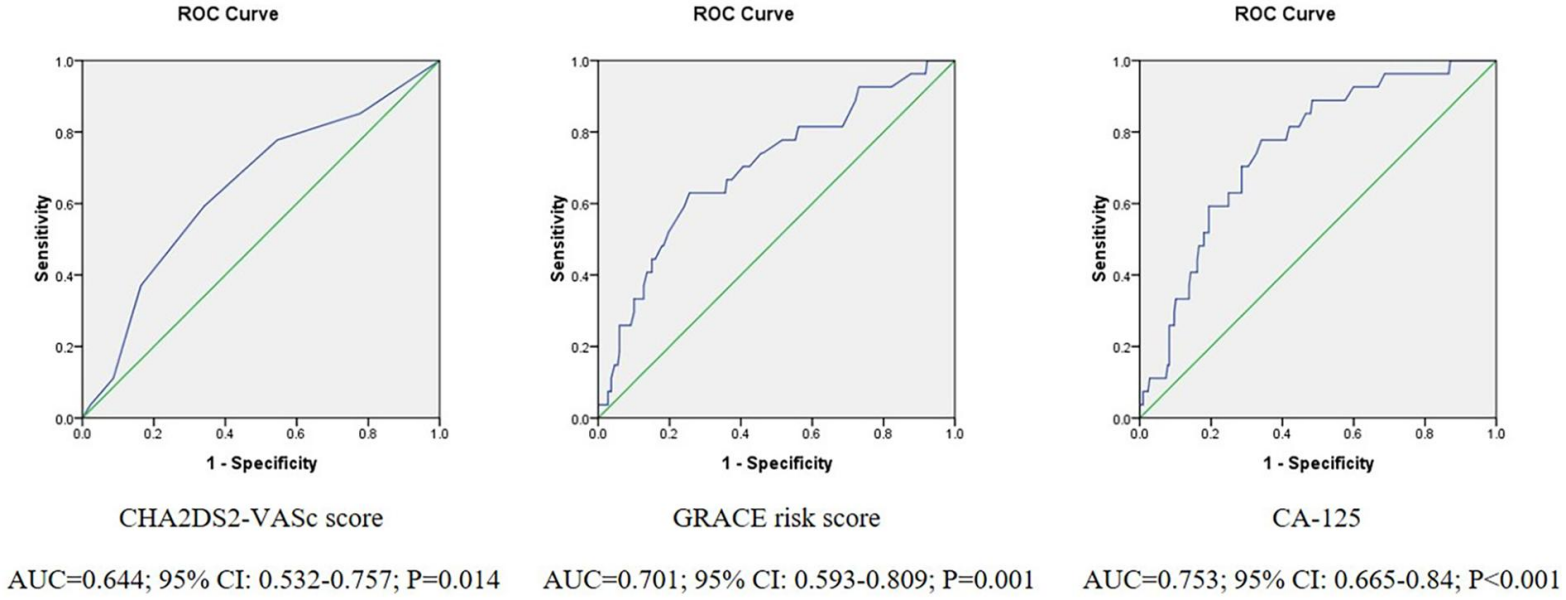
Receiver operating characteristic curve analysis for the CHA2DS2-VASc score, GRACE risk score, and CA-125 for predicting post-AMI NOAF.

There was no statistically significant difference in the CHADS2 score between the patients with and without NOAF (Table 1). The CHA2DS2-VASc score and GRACE RS of the patients with NOAF were significantly higher than those of the patients without NOAF during their hospitalization [4 (3–5) vs. 3 (2–4), *P* = 0.013; 168 (150–190) vs. 150 (130–166), *P* = 0.001; Table 1]. ROC curve analyses were performed and the CHA2DS2-VASc score showed only an acceptable discriminatory power for the prediction of post-AMI NOAF, as evidenced by an AUC = 0.644 (95% CI: 0.532–0.757, *P* = 0.014, Figure 1). However, the discriminatory ability of the GRACE RS for the prediction of NOAF after AMI was relatively high, as evidenced by an AUC = 0.701 (95% CI: 0.593–0.809, *P* = 0.001, Figure 1).

### CA-125 was an independent risk factor for post-AMI NOAF

Pearson correlation analyses were performed and the results showed that CA-125 was negatively correlated with LVEF (*r* = −0.151, *P* = 0.009). There was no significant correlation between CA-125 and the other clinical characteristics, such as white blood cell count (*r* = −0.074, *P* = 0.201), erythrocyte sedimentation rate (*r* = 0.021, *P* = 0.735), high-sensitivity C-reactive protein (*r* = 0.036, *P* = 0.563), BNP (*r* = 0.045, *P* = 0.432), and KILLIP class (*r* = 0.048, *P* = 0.409).

In the univariable analysis, the patients with NOAF had a higher level of CA-125 (Table 1), and a high CA-125 level remained an independent risk factor for post-AMI NOAF in the multivariate logistic regression analyses that included demographic characteristics (age and gender, Table 2, model 1), clinical parameters (BNP and LDL-C, Table 2, model 2), and echocardiography parameters (LVEF, LVESd, and LAD, Table 2, model 3). We performed another multivariate logistic regression analysis, which included the factors in models 1–3 (age, BNP, LDL-C, LVEF, LAD, and CA-125). The results showed that CA-125 was independently associated with post-AMI NOAF (Table 2, model 4). Similar results were found in multivariate logistic regression analyses that included different risk scores (CHA2DS2-VASc score and GRACE RS, Table 2, models 5 and 6). The factors included in these multivariate logistic regression analyses were statistically significantly different between the patients with and without NOAF in the univariable analyses (Table 1). In the ROC analysis, the optimal cut-off level of CA-125 to predict NOAF after AMI was 7.35 U/mL (sensitivity: 77.78%, specificity: 68%, AUC = 0.753, 95% CI: 0.665–0.84, *P* < 0.001, Figure 1).

**Table 2:** Multivariate logistic regression analyses for independent risk factors for post-AMI NOAF.

Variable	Estimated β	OR (95% CI)	*P*-value
Model 1			
CA-125	**0**.**042**	**1.043** (**1.012–1.075)**	**0**.**007**
Age, years	**0**.**08**	**1.083** (**1.034–1.136)**	**0**.**001**
Male	0.013	1.013 (0.341–3.013)	0.981
Model 2			
CA-125	**0**.**031**	**1.032** (**1.008–1.066)**	**0**.**026**
BNP, pg/mL	**0**.**001**	**1.001** (**1–1.002)**	**0**.**019**
LDL-C, mmol/L	-0.402	0.669 (0.417–1.072)	0.095
Model 3			
CA-125	**0**.**036**	**1.037** (**1.003–1.071)**	**0**.**03**
LVEF, %	−0.047	0.954 (0.905–1.006)	0.084
LVESd, mm	−0.003	0.997 (0.902–1.102)	0.959
LAD, mm	0.003	1.003 (0.985–1.021)	0.739
Model 4			
CA-125	**0**.**034**	**1.034** (**1.002–1.068)**	**0**.**038**
Age, years	**0**.**069**	**1.071** (**1.019–1.126)**	**0**.**007**
BNP, pg/mL	0.001	1 (0.999–1.001)	0.663
LDL-C, mmol/L	−0.416	0.66 (0.406–1.071)	0.093
LVEF, %	−0.031	0.97 (0.929–1.012)	0.158
LAD, mm	0.002	1.002 (0.98–1.024)	0.864
Model 5			
CA-125	**0**.**043**	**1.044** (**1.012–1.077)**	**0**.**007**
CHA2DS2-VASc score	**0**.**274**	**1.316** (**1.032–1.677)**	**0**.**027**
Model 6			
CA-125	**0**.**037**	**1.038** (**1.007–1.071)**	**0**.**018**
GRACE RS	**0**.**022**	**1.023** (**1.008–1.038)**	**0**.**003**

NOAF, new-onset atrial fibrillation; CA-125, cancer antigen-125; BNP, B-type natriuretic peptide; LDL-C, low-density lipoprotein; LVEF, left ventricular ejection fraction; LVESd, left ventricular end-systolic diameter; LAD, left atrium diameter; GRACE RS, Global Registry of Acute Coronary Events risk score.

Variables included in model 1 were age and gender. Variables included in model 2 were CA-125, BNP and LDL-C. Variables included in model 3 were CA-125, LVEF, LVESd and LAD. Variables included in model 4 were CA-125, age, BNP, LDL-C, LVEF, LAD. Variables included in model 5 were CA-125 and the CHA2DS2-VASc score. Variables included in model 6 were CA-125 and the GRACE RS.

Bold values indicate *P* value <0.05.

### The comparison of AUCs

**Figure 2:**
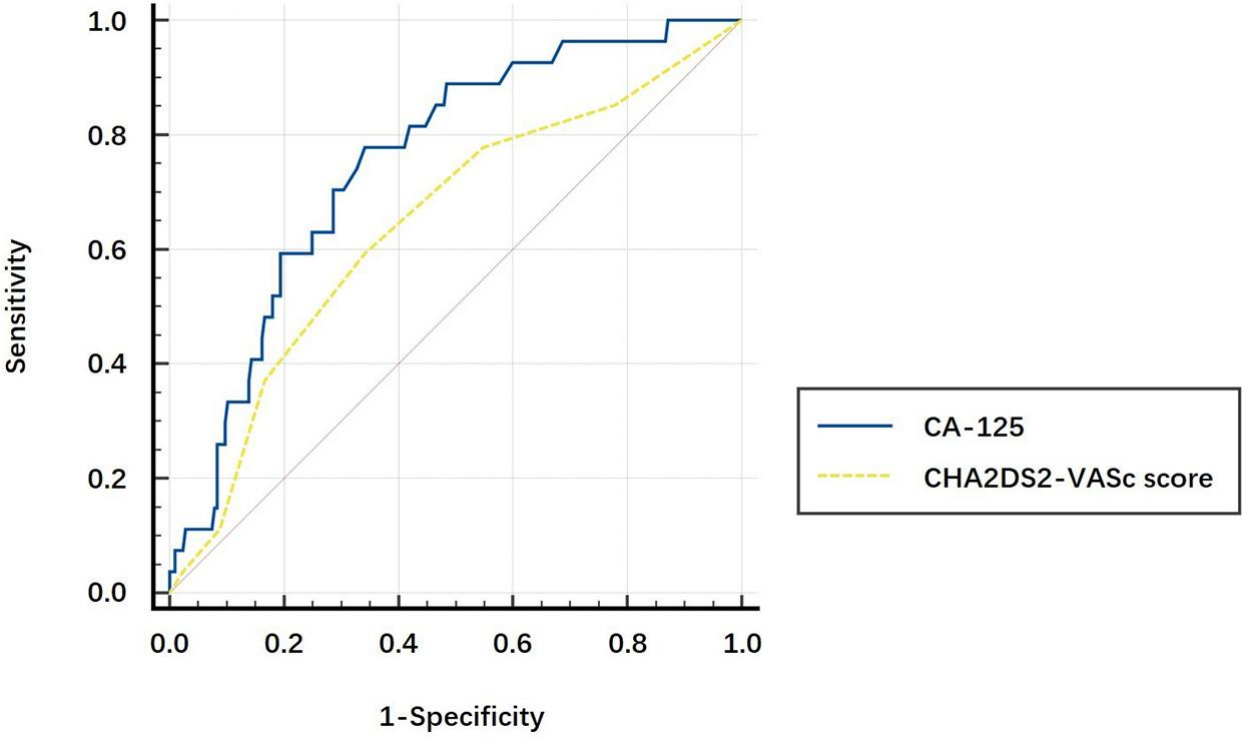
The comparison of the areas under the curve using the *Z* test. The AUC of CA-125 for predicting post-AMI NOAF was significantly higher than that of the CHA2DS2-VASc score (0.753 vs. 0.644, *P* = 0.038).

**Figure 3:**
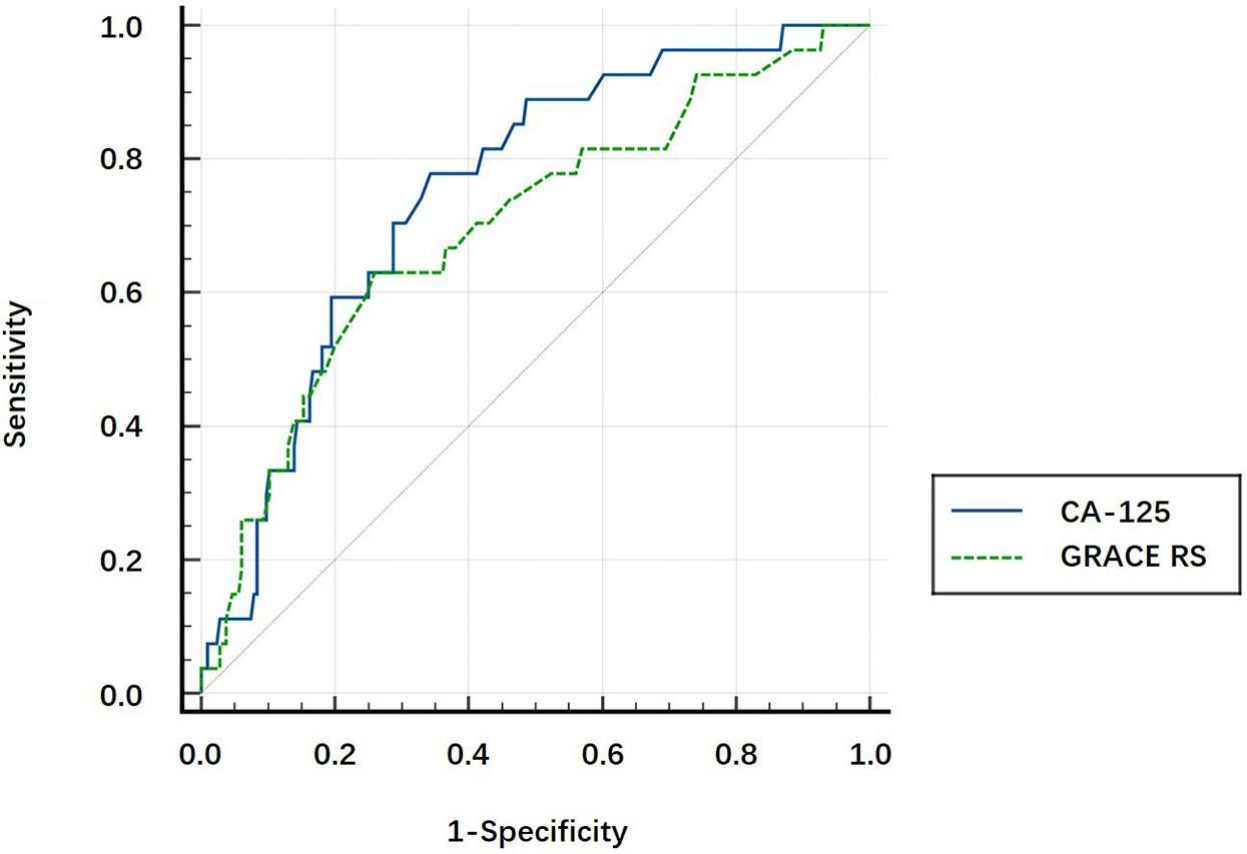
The comparison of the areas under the curve using the *Z* test. The AUC of CA-125 for predicting post-AMI NOAF tended to be higher than that of GRACE RS (0.753 vs. 0.701, *P* = 0.353).

CA-125, the GRACE RS, and the CHA2DS2-VASc score were found to be independent risk factors for post-AMI NOAF; thus, we evaluated the prognostic value of each variable. The AUCs of CA-125, the GRACE RS, and the CHA2DS2-VASc score to predict NOAF after AMI were 0.753 (95% CI: 0.665–0.84, *P* < 0.001), 0.701 (95% CI: 0.593–0.809, *P* = 0.001), and 0.644 (95% CI: 0.532–0.757, *P* = 0.014), respectively. After *Z* tests, the discriminatory ability of CA-125 for the prediction of post-AMI NOAF was significantly higher than that of the CHA2DS2-VASc score (*P* = 0.038, Figure 2), but it was not statistically significantly higher than that of the GRACE RS (*P* = 0.353, Figure 3).

## Discussion

In the present study, a high plasma CA-125 level was found to be an independent risk factor for post-AMI NOAF. The discriminatory ability of CA-125 in predicting NOAF after AMI during hospitalization was relatively high, higher than that of the CHA2DS2-VASc score (*Z* test, 0.753 vs. 0.644, *P* = 0.038), and tended to be higher than that of the GRACE RS (*Z* test, 0.753 vs. 0.701, *P* = 0.353). To the best of our knowledge, this is the first study that demonstrates that CA-125 may be a promising biomarker that can predict post-AMI NOAF during hospitalization in Chinese patients.

The development of NOAF during hospitalization in AMI patients has been extensively proven to be associated with a worse clinical prognosis, such as increased embolism events, congestive heart failure, and short-, mid-, and long-term mortality (1, 2, 21). In our study, the incidence of post-AMI NOAF was 9.84%, which is in line with previous published data (4%–21%) (1, 5, 22). The majority of the NOAF cases (6.97%) developed after the first 24 h after admission. The in-hospital mortality rate among the patients with NOAF was significantly higher than that among the patients without NOAF (10.42% vs. 0.91%, *P* < 0.001). Similar and slightly higher in-hospital mortality rates among patients with NOAF (about 15%) have been found in previous studies (6, 22).

Previous studies have also demonstrated that several factors are associated with post-AMI NOAF, such as age, gender, obesity, HR, Killip class, BNP level, hs-CRP level, LAD, and LVEF (21, 23, 24). However, there are scarce data on the association between post-AMI NOAF and plasma CA-125 level. CA-125 is a high-molecular-weight soluble glycoprotein used for the detection and monitoring of the recurrence of ovarian cancer, with moderate specificity and high sensitivity (25). The typical cut-off level of CA-125 to detect ovarian cancer is >35 U/mL (25). Previous studies have shown an association between CA-125 and cardiovascular diseases, such as HF, coronary artery disease (CAD), and AF. For example, a study by Nunez et al. showed that a higher CA-125 level was positively associated with worse heart function and congestion parameters (26). A study by Rong et al. found a positive association between elevated CA-125 level and the severity of coronary artery disease, and a high CA-125 level was an independent risk factor for mortality in patients with CAD (27). For AF, Kaya et al. showed that a higher CA-125 level was related to the presence of permanent AF in patients with systolic heart failure (8). A study by Sekiguchi et al. demonstrated that CA-125 was a biomarker for NOAF in postmenopausal women (7). A study by Huang et al. reported a positive relationship between a higher plasma CA-125 level and 1-year recurrence of AF after catheter ablation (10). In our study, we demonstrated that a higher CA-125 level was an independent risk factor for post-AMI NOAF with high diagnostic accuracy, and the optimal cut-off level of CA-125 to predict NOAF after AMI was 7.35 U/mL. The best cut-off level of CA-125 to predict NOAF was not consistent in published data (from 9.8 to 91 U/mL). This may be due to the differences in the study population and testing methods and kits for CA-125 (7–9).

The mechanisms for the association between elevated CA-125 levels and NOAF are not well understood. Previous studies have shown that inflammation and mechanical stress in the atrial and ventricular walls play an important role in the development of AF (28–30). In AMI, the resulting myocardial necrosis triggers an inflammatory cascade that, while inherently detrimental, is essential for the clearance of cellular debris and the initiation of tissue repair (31). Although the post-AMI inflammation is, to a degree, necessary for myocardial healing, a prolonged or excessive inflammatory response can be detrimental, leading to chronic contractile dysfunction, hemodynamic stress on atrial/ventricular wall, and adverse myocardial remodeling (31). All these factors may play an important role in the development of NOAF after AMI. CA-125 is produced by coelomic epithelial cells, such as pleural, peritoneal, pericardial, and Müllerian epithelial cells, following mechanical stress and inflammatory stimulation (32). De Gennaro et al. demonstrated that circulating CA-125 levels were related to other inflammatory cytokine levels, such as tumor necrosis factor (TNF), interleukin-6, and interleukin-10. Elevated CA-125 levels may therefore be secondary to cytokine activation (33). A study by Huang et al. showed that mechanical stress and inflammatory stimulation are transmitted into the cytoplasm through the c-Jun N-terminal kinase pathway and cause mesothelial cells to synthesize CA-125. The change in the stability of the cell membrane and the morphocytology of coelomic epithelial cells activate the extracellular domain of the CA-125 that is shed from the cells (34). Moreover, AF can also cause hemodynamic changes, tachycardia, and irregular rhythm, impairing ventricular systolic function, while the loss of atrial systole worsens diastolic function. The consequent increase in atrial/ventricular filling pressure promotes myocardial remodeling and may thereby drive the synthesis of CA-125, resulting in higher plasma levels. Thus, the elevated CA-125 levels observed in patients with NOAF may be associated with more severe congestion and inflammation caused by myocardial necrosis and the following development of NOAF after AMI. According to the Fourth Universal Definition of Myocardial Infarction, T2MI is characterized by acute myocardial injury arising from ischemic events (11). This condition is specifically caused by a mismatch between myocardial oxygen supply and demand, in the absence of an acute atherothrombotic event such as coronary plaque rupture or thrombosis (12, 13). Compared with T1MI, T2MI is usually associated with a poorer prognosis and demonstrates important age- and gender-related differences in clinical presentation and outcomes (13). T2MI represents a distinct pathogenetic mechanism from T1MI, with potentially different inflammatory profiles and arrhythmogenic mechanisms. As a result, we excluded patients with T2MI in this study, as CA-125 may behave differently in this population.

The CHA2DS2-VASc score is used for stroke risk stratification in patients with non-valvular atrial fibrillation and can help physicians in selecting anticoagulation strategies (14). The GRACE RS is a validated risk stratification model used to predict adverse clinical outcomes, such as mortality in patients with ACS, and it has been recommended by current guidelines in the management of patients with ACS (35, 36). Recently, the CHA2DS2-VASc score and GRACE RS were demonstrated to be independent risk factors for post-AMI NOAF during hospitalization (6, 18). Similar results were found in our study: both these scores were valid tools for predicting NOAF after AMI. The AUCs of the CHA2DS2-VASc score and GRACE RS were 0.644 (95% CI: 0.532–0.757, *P* = 0.014) and 0.701 (95% CI: 0.593–0.809, *P* = 0.001), respectively.

The AUC of CA-125 for predicting post-AMI NOAF was the highest (AUC = 0.753, 95% CI: 0.665–0.84, *P* < 0.001) compared with the AUCs of the CHA2DS2-VASc score and GRACE RS. As a clinical biomarker, the half-life of CA-125 was relatively long (ranging from 4 to 21 days with an average of 10 days), and its plasma level is not affected by the age, BMI, or renal function of patients (37). Moreover, as a widely available biomarker, CA-125 was cheaper than other biomarkers, such as BNP (10). Thus, CA-125 showed great potential and advantage in predicting post-AMI NOAF during hospitalization, with a relatively high diagnostic accuracy. It is very convenient to identify patients with AMI who are at higher risk of developing NOAF during hospitalization by measuring plasma CA-125 level, and individualized therapeutic decisions can then be made by clinicians to improve the prognosis of patients. For instance, aggressive statin therapy and the timely initiation of anticoagulant therapy, with an appropriate duration, may be beneficial for patients with AMI with a higher plasma CA-125 level on admission, indicating a higher risk of developing NOAF during hospitalization (14, 38). Sodium-glucose co-transporter 2 (SGLT2) inhibitors have shown a significant improvement in outcomes in a wide range of cardiovascular diseases (39). Beyond their effects on glycemic control and osmotic diuresis, SGLT2 inhibitors confer cardiovascular protection by not only improving endothelial function and vasodilation to optimize myocardial energy metabolism and preserve contractility but also by potentially mitigating inflammation and attenuating ischemia/reperfusion injury. These mechanisms collectively contribute to reduced infarct size, enhanced left ventricular function, and decreased arrhythmia susceptibility (39, 40). The ability of SGLT2 inhibitors to prevent AF has been evaluated in various studies. As a result, the early initiation of SGLT2 inhibitors post-AMI, particularly in patients with elevated CA-125 levels, may be associated with a reduced risk of NOAF and improved overall prognosis (40). However, although CA-125 could serve as a valuable risk stratification biomarker, the absence of evidence for outcome improvement currently may preclude its recommendation for routine testing post-AMI. Individualized management is still mandatory and the beneficial effects should be verified in randomized controlled trials in the future.

## Limitations

There were some limitations in this study. First, as a single-center observational retrospective study with a relatively small sample size, the potential cause-and-effect relationship could not be determined. Second, the plasma level of CA-125 was only measured at baseline and any changes during hospitalization were not measured. Third, there was a complex interplay between AF and cancer, and the impact of occult tumors on CA-125 levels is difficult to accurately estimate (41, 42). Fourth, long-term follow-up was not performed and any possible residual confounding factors could not be determined.

## Conclusions

In this observational study of Chinese patients with AMI, elevated plasma CA-125 levels at admission were significantly associated with an increased risk of NOAF during hospitalization. A CA-125 level >7.35 U/mL demonstrated substantial discriminatory power and was identified as an independent predictor for NOAF in this cohort. These findings suggest an association that warrants further investigation in prospective studies to establish its predictive utility and elucidate any potential causal mechanisms.

## Data Availability

The data analyzed in this study are subject to the following licenses/restrictions: The datasets generated and/or analyzed during the current study are not publicly available due to the restrictions of the human genetics data policy of the Beijing Chaoyang Hospital Ethics Committee, but are available from the corresponding author on reasonable request. Requests to access these datasets should be directed to Lin Zhao at zhaolinl666@163.com.
